# Genome-Wide RNAi Screening Identifies Novel Pathways/Genes Involved in Oxidative Stress and Repurposable Drugs to Preserve Cystic Fibrosis Airway Epithelial Cell Integrity

**DOI:** 10.3390/antiox10121936

**Published:** 2021-12-02

**Authors:** Javier Checa, Itziar Martínez-González, Maria Maqueda, Jose Luis Mosquera, Josep M. Aran

**Affiliations:** 1 Immune-Inflammatory Processes and Gene Therapeutics Group, Genes, Disease and Therapy Program, Institut d’Investigació Biomèdica de Bellvitge—IDIBELL, L’Hospitalet de Llobregat, 08908 Barcelona, Spain; jcheca@idibell.cat (J.C.); imartinez@idibell.cat (I.M.-G.); 2 Department of Experimental Immunology, Amsterdam University Medical Centers, University of Amsterdam, 1012 WX Amsterdam, The Netherlands; 3 Bioinformatics Unit, Institut d’Investigació Biomèdica de Bellvitge—IDIBELL, L’Hospitalet de Llobregat, 08908 Barcelona, Spain; mmaqueda@idibell.cat (M.M.); jmosquera@idibell.cat (J.L.M.)

**Keywords:** oxidative stress, airway epithelial cells, cystic fibrosis, RNAi screening, data mining, drug databases

## Abstract

Recurrent infection-inflammation cycles in cystic fibrosis (CF) patients generate a highly oxidative environment, leading to progressive destruction of the airway epithelia. The identification of novel modifier genes involved in oxidative stress susceptibility in the CF airways might contribute to devise new therapeutic approaches. We performed an unbiased genome-wide RNAi screen using a randomized siRNA library to identify oxidative stress modulators in CF airway epithelial cells. We monitored changes in cell viability after a lethal dose of hydrogen peroxide. Local similarity and protein-protein interaction network analyses uncovered siRNA target genes/pathways involved in oxidative stress. Further mining against public drug databases allowed identifying and validating commercially available drugs conferring oxidative stress resistance. Accordingly, a catalog of 167 siRNAs able to confer oxidative stress resistance in CF submucosal gland cells targeted 444 host genes and multiple circuitries involved in oxidative stress. The most significant processes were related to alternative splicing and cell communication, motility, and remodeling (impacting cilia structure/function, and cell guidance complexes). Other relevant pathways included DNA repair and PI3K/AKT/mTOR signaling. The mTOR inhibitor everolimus, the α1-adrenergic receptor antagonist doxazosin, and the Syk inhibitor fostamatinib significantly increased the viability of CF submucosal gland cells under strong oxidative stress pressure. Thus, novel therapeutic strategies to preserve airway cell integrity from the harsh oxidative milieu of CF airways could stem from a deep understanding of the complex consequences of oxidative stress at the molecular level, followed by a rational repurposing of existing “protective” drugs. This approach could also prove useful to other respiratory pathologies.

## 1. Introduction

Respiratory dysfunction and failure have devastating consequences in cystic fibrosis (CF), the most frequent hereditary autosomal disease in the Caucasian population. CF is caused by mutations in the *CFTR* gene leading to absence or abnormal function of the CFTR anion channel in the surface of epithelial cells, although its pathophysiology remains incompletely understood. Failure of chloride and bicarbonate secretion and concomitant sodium hyperabsorption at the airway apical surface induces dehydration of the superficial fluid layer and impairs mucociliary clearance [1,2,3]. These events support muco-obstruction and airborne bacterial infection [4,5], which activate the innate airway defenses in the host, starting a positive feedback cycle between chronic inflammation and acute exacerbations and evolving to irreversible airway destruction and fibrosis [6].

Neutrophils first and alveolar macrophages later, main effectors in the innate immune response, in an attempt to clear pathogens and resolve airway inflammation at the site of infection, generate supraphysiological levels of reactive oxygen species (ROS), paradoxically contributing to oxidative stress, apoptosis, and structural lung damage. This is particularly serious in CF patients, which exhibit disrupted airway epithelium homeostasis and major deficiencies in the antioxidant and detoxification systems, such as an impaired efflux of reduced glutathione, leading to increased oxidative cross linkage of mucins through disulfide bonds [7]. Moreover, recent studies suggest that certain bacteria can also produce H_2_O_2_ [8]. CF patients experience also malabsorption of dietary antioxidants, particularly those with pancreatic insufficiency [9]. Nevertheless, antioxidant supplementation aimed to prevent the extent of oxidative lesions in CF lung disease has not met expectations up to now in terms of a clearly favorable clinical outcome, although oral glutathione appeared to show some benefit improving lung function and decreasing oxidative stress [10]. Therefore, the possibility of reducing the impact of ROS in the host’s airway epithelia might have a huge influence on lung function and on the progression of CF. In fact, H_2_O_2_-mediated oxidative stress induces a plethora of epithelial cell-specific responses including endoplasmic reticulum stress, which could end up causing DNA damage and apoptosis through activation of mitogen-activated protein kinases, translocation of the NF-κB complex into the nucleus, and inflammasome activation. Furthermore, the lung holds structural and functional airway epithelial cell diversity and, being constantly exposed to infection and other environmental stresses, shows lineage plasticity in response to injury [11]. Consequently, the detailed molecular mechanisms and the full spectrum of biological pathways involved in ROS-mediated actions over airway epithelial cells is complex and has not yet been fully defined. Through genome-wide RNAi screening using a randomized siRNA library, we set out to identify novel genes, pathways, and cues involved in oxidative stress susceptibility in CF airway submucosal gland cells. Under physiological conditions, these cells produce the mucus that lines the upper airways to protect them against insults, such as infection, smoke, or other inspired substances known to induce oxidative stress and persistent inflammation. Further siRNA target data mining led to the identification of several approved drugs able to confer significant oxidative stress resistance and prevent cell death in CF airway epithelial cells. These drugs could, therefore, be successfully repurposed for the treatment of CF airway pathology and other chronic airway disorders in which oxidative stress plays a key role.

## 2. Materials and Methods

### 2.1. Cell Culture

HEK 293 (human epithelial kidney cells), HCT116 (human colon carcinoma epithelial cells), and U2OS cells (human bone osteosarcoma epithelial cells), all from ATCC (Manassas, VA), were used for the characterization of the convergent expression system. HEK293 and U2OS cells were grown in DMEM, and HCT116 cells were grown in RPMI1640 (all from Thermo Fisher, Walthman, MA, USA). All of them were supplied with 2 mM glutamine, penicillin (100 U/mL), streptomycin (100 μg/mL), and 10% FBS. For optimization of siRNA expression into airway epithelial cells, three different human cell lines were managed: 16HBE14o-, CFBE41o-, and 6CFSMEo- [12]. All of them were kindly provided by Dieter Gruenert (UCSF, San Francisco, CA, USA) and maintained in MEM with Earle’s salts (Thermo Fisher) supplemented with 2 mM glutamine, penicillin (100 U/mL), streptomycin (100 μg/mL), and 10% FBS. The 6CFSMEo- submucosal gland epithelial cells [13,14] were further employed for siRNA library transfection, selection, and characterization of oxidative stress-resistance. All cells were grown at 37 °C and 5% CO_2_.

### 2.2. Construction of a Convergent siRNA Expression System

To efficiently induce RNAi-mediated gene silencing into the abovementioned epithelial cells, symmetric transcription was employed using a convergent human RNA polymerase III (Pol III)-based promoter cassette [15] and its functionality was checked through expression of an siRNA against the reporter gene EGFP. Two different promoters were exploited, U6 and H1, to avoid inverted repeats leading to plasmid instability when introduced in *E. coli*. Thus, the U6 promoter was first amplified followed by the siRNA against EGFP from the pGEM-U6 plasmid using primers ClaI/NotI F and BamHI-MluI-siRNA(EGFP)-SmaI R (Table S1).

**Figure 1:**
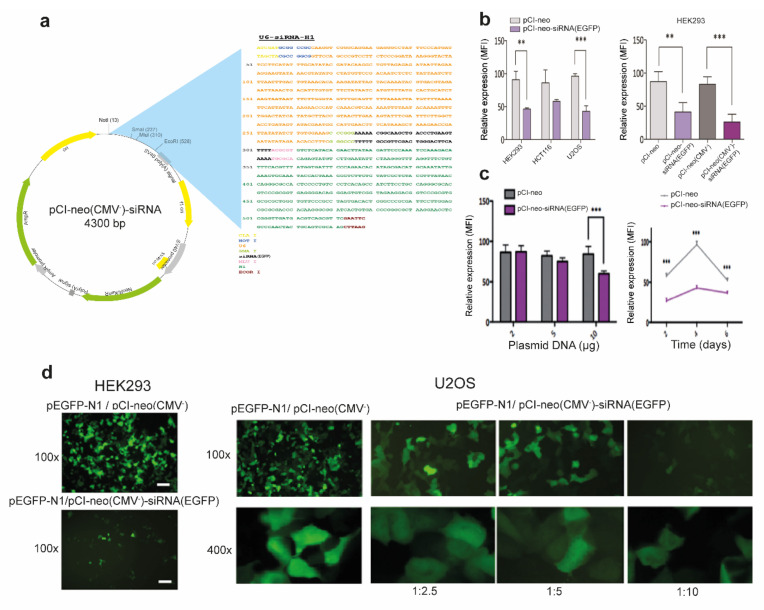
Functional validation of pCI-neo(CMV^−^)-siRNA knock-down efficacy in epithelial cells. (**a**) Scheme of the pCI-neo(CMV^−^)-siRNA expression vector. The upper right side shows a detail of the cloned convergent expression cassette holding the U6 and H1 polymerase III promoters in opposite orientation substituting the CMV promoter. (**b**) Functional characterization of the convergent expression system for siRNA production. Left: co-transfection of pEGFP-N1 and pCI-neo or pCI-neo-siRNA(EGFP) (1:5) in HEK293, HCT116 and U2OS cells. Right: co-transfection of pEGFP-N1 and pCI-neo, pCIneo-siRNA(EGFP), pCI-neo(CMV-), or pCI-neo(CMV-)-siRNA(EGFP) (all at 1:5 ratio) in HEK293 cells. (**c**) Dose-response assay. Left: transfection of different amounts of pCI-neo(CMV-) or pCIneo(CMV-)-siRNA(EGFP) (2–10 μg) within clone 293FT-EGFP. Right: co-transfection of pEGFP-N1 and pCI-neo(CMV-) or pCI-neo(CMV-)-siRNA(EGFP) in 293FT-EGFP cells analyzed at various times post-transfection. In all cases the median fluorescence intensity (MFI) was analyzed by flow cytometry. Data are expressed as means ± SD. *n* = 3 with 4–6 technical replicates/each. **p < 0.01; ***p < 0.001 compared to the control. (**d**) Representative fluorescence microscopy images showing the efficiency of the siRNA convergent expression system in HEK293 and U2OS cells. At 48 h after co-transfecting pEGFP-N1 and pCI-neo(CMV-) or pCI-neo(CMV-)-siRNA(EGFP) at the indicated ratios and magnifications.

The corresponding, MluI/ClaI-digested 320 bp fragment was cloned into the analogously digested lentiviral vector pLVTHM, which co-expresses shRNAs under the H1 promoter and the reporter EGFP under the EF1α promoter (kindly supplied by Didier Trono, Laussane, Switzerland), leading to pLVTHM-U6-siRNA(EGFP). The corresponding convergent expression module, 5′-ClaI-NotI-U6-SmaI-siRNA(EGFP)-MluI-H1-EcoRI-3′, was also excised from pLVTHM-U6-siRNA(EGFP) with NotI/EcoRI and inserted in the analogously digested pCI-neo expression vector (Promega, Madison, WS) yielding pCIneo-U6-siRNA(EGFP)-H1 (pCIneo-siRNA(EGFP)). Finally, to prevent promoter interference potentially affecting the convergent expression module within pCIneo-siRNA(EGFP), the CMV promoter was deleted by BglII/XhoI digestion, Klenow fill-in, and re-ligation, obtaining pCI-neo(CMV^−^)-siRNA(EGFP) (Figure 1a).

### 2.3. Plasmid Transfections

To assess the performance of the convergent siRNA expression cassette, pLVTHM-U6-siRNA(EGFP), pCI-neo-siRNA(EGFP), and pCI-neo(CMV^−^)-siRNA(EGFP) expression vectors were transfected into 60% confluent epithelial cell lines using different vehicles (Ca_3_(PO_4_)_2_, polyethylenimine (PEI) (jetPEI, Polyplus transfection, Illkirch, France), and polyfect (Qiagen, Hilden, Germany)) with similar efficiencies. Three different strategies were employed: (1) co-transfection of pEGFP-N1 (Clontech, Mountain View, CA), expressing the reporter gene EGFP (1 μg) + 5 μg pCI-neo-siRNA(EGFP), or co-transfection of pEGFP-N1 (Clontech) (1 μg) + 5 μg pCI-neo(CMV^−^)-siRNA(EGFP); (2) transfection of different amounts of pCI-neo(CMV^−^)-siRNA(EGFP) (0.5 μg–10 μg) into a HEK293FT clone constitutively expressing high levels of EGFP after transduction with the pWPT-GFP lentiviral vector (also provided by Didier Trono); and (3) transfection of pLVTHM-U6-siRNA(EGFP) (5 μg). In all instances, the corresponding expression vectors pCI-neo, pCI-neo(CMV^−^), or pLVTHM were used as negative controls. The percentage and intensity of fluorescence from the transfected cells was analyzed by fluorescence microscopy and quantified at different times post-transfection in a FACS-Canto II flow cytometer equipped with CellQuestPro software (Becton Dickinson, Franklin Lakes, NJ, USA).

Low toxicity polymer-based transfection into the airway epithelial cell lines was optimized using the pEGFP-N1-reported plasmid (Clontech) complexed with TransIT-2020 (Mirus Bio, Madison, WI, USA) according to the manufacturer’s instructions, and at different DNA:cell concentration ratios. Cell fluorescence was analyzed at 36 h after transfection through flow cytometry and/or fluorescence microscopy.

### 2.4. Cell Death Induction through Oxidative Stress

H_2_O_2_ was employed as a source of oxygen-derived free radicals to explore the oxidative stress susceptibility of 6CFSMEo- cells. Thus, 6CFSMEo- cells were exposed to H_2_O_2_ concentrations ranging from 0 to 0.6 mM for different times (1 to 7 days), and their viability was assessed through dose-toxicity curves and live cell counting using a hemocytometer.

### 2.5. Randomized siRNA Library Generation

A genome-wide randomized siRNA library targeting the whole human transcriptome was generated using the above-mentioned convergent expression cassette, including human U6 and H1 Pol III promoters in opposite orientations co-expressing the sense and anti-sense siRNA strands. These promoters were modified to include a 5T polymerase III termination signal between the −5 to −1 position of the promoters and the SmaI and MluI restriction sites [16] (Figure 1a). Thus, using a 2300 bp MluI/BamHI fragment from pCI-neo(CMV^−^)-siRNA(EGFP) as a template, a 732 bp fragment was amplified with “pCIneo(-cmv) LIB F” and “pLIB-N R primers” (Table S1). This last 73-mer primer contains a “randomized” G+N18-nucleotide stretch, yielding a theoretical library “capacity” of 4^18^ (~6.8 × 10^10^) possible siRNAs. Further digestion with XmaI/EcoRI yielded a 254 bp fragment, which was employed to replace an analogous fragment containing the siRNA against EGFP in pCI-neo(CMV^−^)-siRNA(EGFP), generating pCI-neo(CMV^−^)-LB. Additional transformation of pCI-neo(CMV^−^)-LB into electrocompetent *E. coli* DH5α cells (Thermo Fisher) yielded a final amplified library with a titration of 7.1 × 10^9^ cfu/mL.

To analyze siRNA variability and to rule out any sequence bias introduced during the synthesis steps, 30 individual clones from the final library were sequenced (STAB VIDA, Caparica, Portugal), indicating 85% correct full-length siRNAs and 49% G+C content.

### 2.6. Randomized siRNA Library Transfection and Screening

The random siRNA library generated previously was introduced to 6 × 10^7^ exponentially growing 6CFSMEo- cells in suspension by transfection using TransIT-2020 (Mirus Bio) and 30 μg of pCI-neo(CMV^−^)-LB or the control plasmid pCI-neo(CMV^−^). Transfected cells were plated in 37 × 100 mm plates (2 × 10^6^ cells/plate) and at 36 h after transfection the complete MEM was replaced by fresh complete MEM supplemented with 0.3 mM H_2_O_2_, which was lethal in the control pCI-neo(CMV^−^)-transfected plates. The remaining oxidative stress-resistant clones surviving the harsh H_2_O_2_ selection conditions from the pCI-neo(CMV^−^)-LB-transfected plates were further isolated, their genomic DNA was extracted and the siRNA-encoding cassettes with identical flanking sequences were amplified by proofreading PCR using the “ClaI/NotI F” and “EcoRI-H1 R” primers (Table S1) and cloned into the pJET1.2 cloning vector. The cloned pJET-siRNA encoding cassettes were transformed into *E. coli*. Individual transformants were randomly picked for sequencing (STAB VIDA) using “pJET 1.2 F” and “pJET 1.2 R” primers (Table S1). Finally, after three rounds of transfection using the mixed pool of cloned plasmids and H_2_O_2_ selection into 6CFSMEo- cells, 181 different siRNA sequences were obtained.

### 2.7. Validation of siRNAs and Drugs Conferring Oxidative Stress Resistance

A secondary high-throughput screen using a reverse transfection protocol was undertaken to confirm that the 181 siRNAs obtained in the primary screen were able to individually confer H_2_O_2_ resistance in 6CFSMEo- cells. Briefly, 2 × 10^5^ freshly passaged 6CFSMEo- cells were added to pre-plated transfection complexes containing 200 ng/well of convergent expression plasmid DNA, including each of the 181 different siRNA sequences and 2 μL TransIT-2020/μg DNA per well of a 96 well plate (220 μL/well). The pCI-neo(CMV^−^)-siRNA(EGFP) plasmid was used as negative control for selection. Four replicates were performed per siRNA. At 48 h after transfection, the medium from each well was replaced by fresh complete medium supplemented with 0.3 mM H_2_O_2_.

Additionally, knock-down efficiency and capacity to confer oxidative stress resistance from commercial siRNA pools (ON-TARGETplus siRNAs in SMARTpool format, Dharmacon, Lafayette, CO, USA) directed against selected siRNA target genes was verified in 6CFSMEo- cells by transient transfection according to the manufacturer’s indications. Briefly, 3.4 μL (2.0 μM) of siRNA pools directed against TNFRSF1B and KCNQ1OT1 transcripts (Table S1) and a control non-targeting siRNA pool were mixed with 7.5 μL of TransIT-X2 (Mirus Bio) in 250 μL non-supplemented MEM. The siRNA:TransIT-X2 complexes were generated at room temp. for 20 min, distributed dropwise on top of 60–80% confluent 6CFSMEo- cells in wells from a 6-well plate, and incubated for 48 h until further analysis.

On the other hand, the performance of selected candidate drugs conferring oxidative stress resistance obtained by data mining was also assessed. Thus, 5 × 10^3^ 6CFSMEo- cells per well were added in a 96-well plate to a final volume of 100 μL/well. After 24 h, the selected drugs: everolimus, doxazosin (both from Tocris Bioscience, Bristol, UK), fostamatinib (Invivogen, San Diego, CA, USA), and zanubrutinib (MedchemExpress, Monmouth Junction, NJ, USA) were incubated at the indicated concentrations for 24 h according to preliminary optimization assays. Consequently, the cells were exposed to 100 μL/well of 0.9 mM H_2_O_2_ plus fresh drugs. As a negative control, cells without drug treatment exposed to 0.9 mM H_2_O_2_ were used. Conversely, as a positive control, cells treated with the above drugs but without H_2_O_2_ exposure confirmed its lack of toxicity at the concentrations tested.

In both the siRNA and the drug assays, after 6 h of cell exposure to H_2_O_2_, the oxidant was removed and cell viability monitorization was initiated by incubation with Alamar Blue reagent (100 μL/well in complete medium) (Bio-Rad, Hercules, CA, USA). Spectrophotometer readings were made at 19 h, 24 h, 48 h, 72 h, 120 h, and 144 h after Alamar Blue addition and the survival rate of the 6CFSMEo- cells was assessed according to the manufacturer’s instructions.

### 2.8. Differential Gene Expression Analysis

Total RNA from CF airway epithelial cells was extracted using the RNeasy Mini Kit (Qiagen, Hilden, Germany). Selected gene transcripts were further validated by RT-qPCR using the corresponding inventoried TaqMan Gene Expression Assays (Applied BioSystems). Quantification was achieved through the ΔΔCt method. A relative fold change in mRNA abundance was calculated with the equation 2^−ΔΔCt^, employing cyclophilin A (peptidylprolyl isomerase A (PPIA)) as an endogenous reference transcript.

### 2.9. Identification of Putative siRNA Targets

To identify candidate siRNA targets, sequence homology searches were conducted online using the *blastn* program on NCBI BLAST web site [17]. Searches were driven against human genomic and transcript databases using highly similar sequences optimization (megablast) [18]. Those obtained hits per siRNA query sequence showing the lowest E-value were considered as candidate siRNA targets. Only those hits referring to mRNA, RNA, or protein molecule types were kept. Based on this, hits list was reduced to those with accession number prefix *NM*, *NR*, *NP*, *XM*, *XR*, or *XP*. Gene symbols and Entrez Gene identifiers were annotated based on the corresponding annotations file retrieved from https://ftp.ncbi.nih.gov/gene/DATA/ on the 27 May 2020. Hits with no Entrez Gene identifier annotation were removed. Duplicated gene symbols were reduced to the one with the minimum E-value per siRNA query sequence.

### 2.10. Protein-Protein Interaction (PPI) Networks

In-house R-scripts (R v3.6.0) [19] were used for building protein-protein interaction (PPI) networks based on the STRING database (v11.0) [20]. For this purpose, all human protein interactions and corresponding annotations were retrieved from the database website (https://string-db.org) (last accessed: 9 March 2020). An overall network of 445,816 interactions among 16,450 proteins was available with interaction scores ranging from 41 to 998. These interactions exclusively referred to experimental evidence, being excluded transferred-experimental data from other organisms. Entrez Gene identifiers were used to map siRNA target transcripts to STRING database protein identifiers. Starting with the list of siRNA target transcripts, three different PPI networks were built by: (i) including 1st shell interactors, which are genes that directly interact with the targets. A minimum interaction score of 900 was required, considered as a very high confident interaction according to STRING database, (ii) same as the above, but with a minimum interaction score of 700 (high confidence), and (iii) including 1st and 2nd shell interactors, which are genes that indirectly interact with the targets through 1st shell elements. Only very high confidence interactions were deemed due to the potential network large size. Largest Connected Components (LCC), which refer to the largest cluster of connected genes, were identified per obtained PPI. Network analysis and plotting was conducted by means of igraph R package (v1.2.5) [21].

### 2.11. Pathways Over-Representation Analysis (ORA)

To put in biological context the gene lists of interest, an ORA was conducted by means of clusterProfiler R package (v3.14.3) [22]. ORA was independently computed over Kyoto Encyclopedia of Genes and Genomes (KEGG), Reactome, and The Gene Ontology (GO) knowledgebases [23,24,25]. Respective Entrez Gene identifiers were considered. For each queried biological pathway or GO term, a *p*-value was calculated using a hypergeometric distribution test. Multiple testing was controlled by adjusting the Benjamini-Hochberg False Discovery Rate (FDR) [26]. An adjusted *p*-value lower than 0.05 was considered as statistically significant. The background distribution was defined by all available annotations in the relevant knowledgebase.

Additionally, ClueGO application (v2.5.6) for the Cytoscape plug-in (v3.7.2) [27,28] was explored to identify similar functional groups of significant pathways and terms simultaneously integrated from KEGG, Reactome and GO databases. For this purpose, significant terms were first identified by an enrichment hypergeometric test (FDR adjusted *p*-value < 0.05). Grouping was achieved according to kappa score with a threshold set to 0.5. GO terms fusion was enabled. The rest of the parameters were left as default.

### 2.12. Drug Database Mining

For drug database mining, the complete DrugBank (DB) database was downloaded from https://www.drugbank.ca on 21 July (v5.1.7) after access was granted [29]. DB included 3235 human drug target genes associated to 6260 drugs, where 2280 were approved drugs. Therapeutic Targets Database (TTD) information was downloaded from http://db.idrblab.net/ttd/ on 1 September (no specific version) [30]. It included 1222 human drug target genes associated to 13,998 drugs, where 897 were approved drugs. Identified siRNA target genes and corresponding direct interactors were searched into the corresponding database as drug target genes. From the retrieved hits, the respective approved drugs were listed together with the available information.

### 2.13. Statistical Analysis

The capacity to confer resistance to oxidative stress was assessed for each of the siRNA sequences obtained in the initial screen through descriptive statistics. In fact, the cell survival outcomes of test, positive control, and negative control siRNAs at different times were graphed through box plots. Using a mixed-effects model, an analysis of repeated measures was performed to compare the values obtained from each siRNA at the different times with the corresponding values of the negative control. The effect size was calculated applying the Cohen delta with a confidence interval of 95%. Statistical analysis was conducted using the R programming language (v3.4.0). All other data were analyzed through the GraphPad Prism 6.00 software (GraphPad Software Inc., San Diego, CA, USA). Differences between groups were analyzed using parametric (*t* test, ANOVA) or non-parametric (Wilcoxon, Freidman) tests depending on the normality distribution of the data. Unless otherwise stated, data are represented as average values (mean ± SD). In all cases, a value of *p* < 0.05 was considered statistically significant.

## 3. Results

### 3.1. Efficient RNAi-Mediated Transcript Knockdown in Epithelial Cells through Convergent Transcription from RNA Polymerase III Promoters

To generate a powerful and efficient system for high-throughput phenotypic screening, we turned to RNAi-mediated post-transcriptional silencing using convergent transcription [15]. Thus, we generated an expression cassette including the U6 and H1 Pol III promoters placed in opposite orientations and incorporated it into the widely employed pCI-neo mammalian expression vector in which the CMV promoter was deleted to avoid interference with the U6 and H1 promoters comprising the convergent expression module (Figure 1a). To validate the performance of this construct, we cloned between both Pol III promoters a 19 nt sequence previously reported to efficiently silence the reporter EGFP [15]. Thus, both flow cytometry and fluorescence microscopy confirmed that symmetric transcription from convergent Pol III promoters induced efficient, dose-dependent EGFP expression knockdown in several epithelial cell lines (Figure 1b–d).

### 3.2. Genome-Wide Screening Using a Randomized siRNA Library Uncovers siRNA Sequences Able to Confer Oxidative Stress Resistance in CF Airway Epithelial Cells

**Figure 2:**
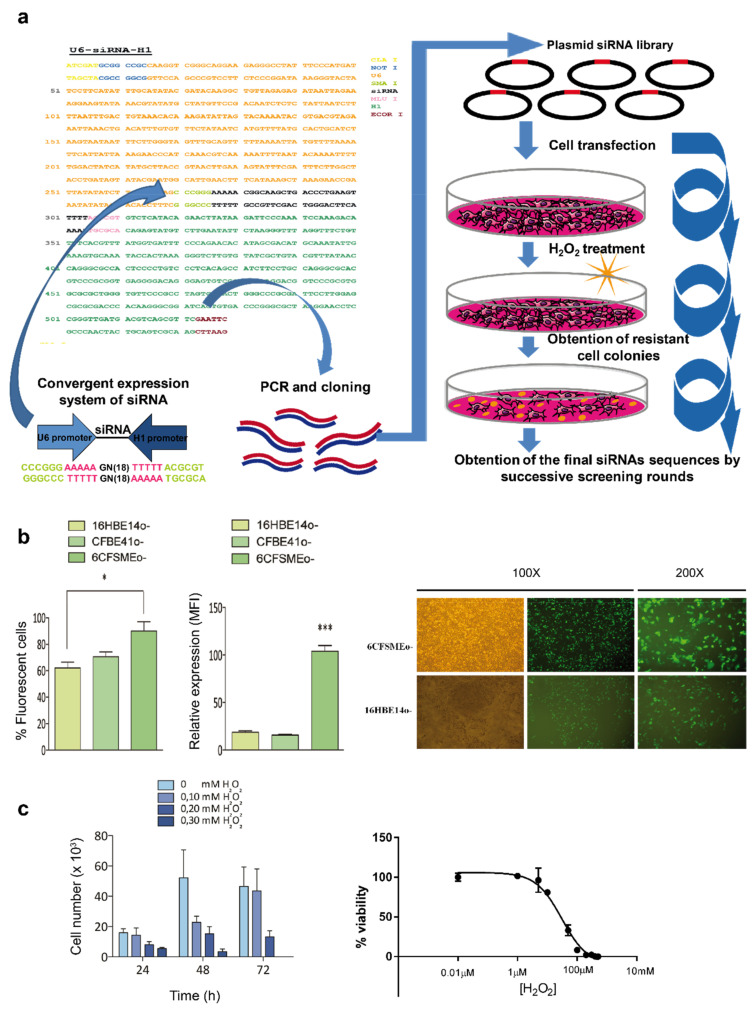
Outline of randomized siRNA library cloning, epithelial cell introduction, and selection of oxidative stress-resistant colonies. (**a**) Schematic representation of the cloning and transfection steps used to obtain the 181 final siRNA sequences. First, a construction containing a randomized siRNA library within a convergent expression cassette was cloned into the pCI-neo vector. The convergent expression system was optimized to express the siRNAs using two opposite promoters. Furthermore, the siRNA library within the convergent expression system was transfected and expressed in CF submucosal gland cells. These cells were subjected to H_2_O_2_-mediated stress and different resistant colonies arose. Successive screening rounds were performed until a final collection of 181 siRNA sequences capable to confer oxidative stress resistance were obtained. (**b**) Optimization of transfection in human airway epithelial cells. Transfection efficiency of human airway epithelial cells. Left: 1 × 10^6^ cells were transfected with 3 μg of pEGFP-N1 and the percentage of fluorescent cells and median fluorescence intensity (MFI) were analyzed by flow cytometry. Data are expressed as means ± SD. *n* = 3 with 5 technical replicates/each. * *p* < 0.05; *** *p* < 0.001 compared to the control. 16HBE14o-, bronchial epithelial cells; CFBE41o-, bronchial epithelial cells homozygous for the *CFTR* F508del mutation; 6CFMEo-, compound heterozygous submucosal gland epithelial cells for the *CFTR* F508del, and Q2X mutations. Right: representative phase contrast and fluorescence microscopy images comparing the transfection efficiencies from 6CFSMEo- and 16HBE14o- cells. (**c**) Assessment of oxidative stress sensitivity in 6CFSMEo- airway epithelial cells. 6CFSMEo- viable cell number trough toxicity assays after exposure to H_2_O_2_. *n* = 4. Left: optimization of the necessary dose of H_2_O_2_ to obtain 90%–100% cell death in 6CFSMEo- cells. At 24 h after seeding 1 × 10^6^ cells per plate, the medium was changed to fresh medium containing different H_2_O_2_ concentrations. Cells were counted at 24, 48, and 72 h. Right: Toxicity curve of H_2_O_2_ in 6CFSMEo- cells. The concentration range used in the study was 0–500 μM.

We devised an unbiased transcriptome-wide siRNA-mediated knockdown strategy followed by stringent selection using strong oxidant conditions to identify novel airway epithelial cell-specific genes and pathways holding an essential role in oxidative stress (Figure 2a). Therefore, a randomized library was constructed (see “Methods” for details) and obtained with a high titer (7.7 × 10^8^ cfu/μg plasmid DNA) to achieve robust, high-throughput knockdown of the whole transcriptome in CF airway epithelial cells, which are particularly prone to oxidative stress-mediated damage and destruction. In fact, the redox imbalance due to the bacterial infection-neutrophil inflammation vicious cycle occurring in the lungs of CF patients holds a key role in the development of the disease [31].

In parallel, a comparative transfection optimization study was undertaken using the reporter vector pEGFP-N1 and three different airway cell lines: non-CF 16HBE14o- bronchial epithelial cells, CFBE41o- bronchial epithelial cells (homozygous CFTR F508del), and 6CFSMEo- submucosal gland epithelial cells (compound heterozygous CFTR F508del/Q2X). Both flow cytometry and fluorescence microscopy indicated that 6CFSMEo- cells achieved the highest transfection efficiency, superior transgene expression, and an optimal proliferation rate (Figure 2b) and, therefore, were selected to introduce the randomized siRNA library. Furthermore, different dose-response assays revealed that 0.3 mM H_2_O_2_ was lethal to 6CFSMEo- cells (Figure 2c).

The initial randomized siRNA library introduction and further screening unveiled several H_2_O_2_-resistant 6CFSMEo- colonies. Due to the high transfection efficiency of 6CFSMEo- cells, each transfectant could have incorporated multiple copies of the siRNA-bearing plasmids. Thus, three successive rounds of transfection-selection, genomic DNA isolation, convergent expression cassette amplification, cloning, and sequencing were necessary to obtain up to 181 unique siRNAs sequences capable to avoid cell death induced by strong oxidative stress.

**Figure 3:**
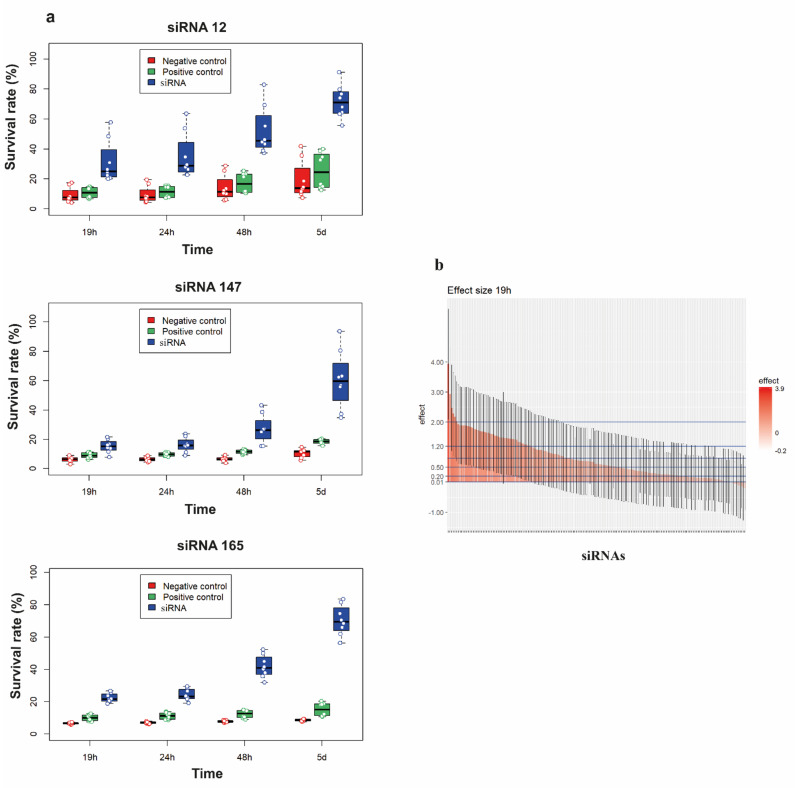
High-throughput validation of the siRNA set conferring oxidative stress resistance to airway epithelial cells. (**a**) Representative spectrophotometric quantification of the mean survival rate of representative test siRNAs (clones 12, 147, and 165; blue), a negative control siRNA (red) and a weak positive control siRNA (clone 171; green) in 6CFSMEo- cells at 19 h, 24 h, 48 h, and 5 days after Alamar Blue reagent addition. *n* = 8 technical replicates/sample. (**b**) Output of the high-content siRNA screening for oxidative stress resistance in airway epithelial cells. The siRNA sequences examined at 19 h (25 h after oxidative stress induction) could be partitioned into those having large effect size (>0.8), medium effect size (between 0.5 and 0.8), small effect size (between 0.2 and 0.5), very small effect size (between 0.01 and 0.2), and no effect size (<0.01). There were 73 siRNAs with a large effect size, 30 siRNAs with a medium effect size, 39 siRNAs with a small effect size, 29 siRNAs with a very small effect size, and 8 siRNAs with no effect.

To validate the individual activity of the 181 siRNA sequences, we re-screened the corresponding siRNA-containing plasmids through a high-throughput and fast functional assay in 96-well plates using reverse transfection of 6CFSMEo- cells, short-term exposition to a lethal H_2_O_2_ concentration, and viability monitorization at different times after high intensity oxidative stress induction. Most unique siRNA-bearing plasmids from the initial screen were able to increase the survival rate of 6CFSMEo- cells challenged with 0.3 mM H_2_O_2_ compared with pEGFP-N1-transfected 6CFSMEo- control cells (Figure 3a). Statistical validation of the assay outcome indicated a moderate variability of the different siRNA clones conferring increased cell survival upon H_2_O_2_ challenge. When the siRNA clones were represented as effect size, where a major effect size represents a higher resistance to oxidative stress, the results obtained were robust and reproducible throughout all times analyzed (Figure 3b). Ultimately, 167 distinctive siRNAs (92%), able to confer significant oxidative stress resistance in airway epithelial cells, were selected for further analysis (Table S2).

### 3.3. Identification of Novel Genes and Pathways Involved in Oxidative Stress in CF Airway Epithelial Cells

To unambiguously identify the siRNA target genes involved in oxidative stress in CF airway epithelial cells a homology BLAST search was conducted per siRNA sequence. This search accurately retrieved 451 annotated target genes, which referred to 444 unique Entrez Gene IDs assigned to 417 protein coding genes, 24 lncRNAs, 2 pseudogenes, and 1 TEC, and distributed throughout all chromosomes (Supplementary Info S1). Surprisingly, only 4 among the 444 unique Entrez Gene IDs (about 1%) were annotated to oxidative stress GO term (GO:0006979): *CHD6* (chromatin remodeler and key regulator of the oxidative DNA damage response) [32], *RBM11* (oxidative stress-responsive splicing regulator) [33], *MDM2* (protooncogene and regulator of the p53 response to oxidative stress) [34,35], and *SOD2* (mitochondrial MnSOD enzyme playing a key antioxidative role in the lung) [36].

**Figure 4:**
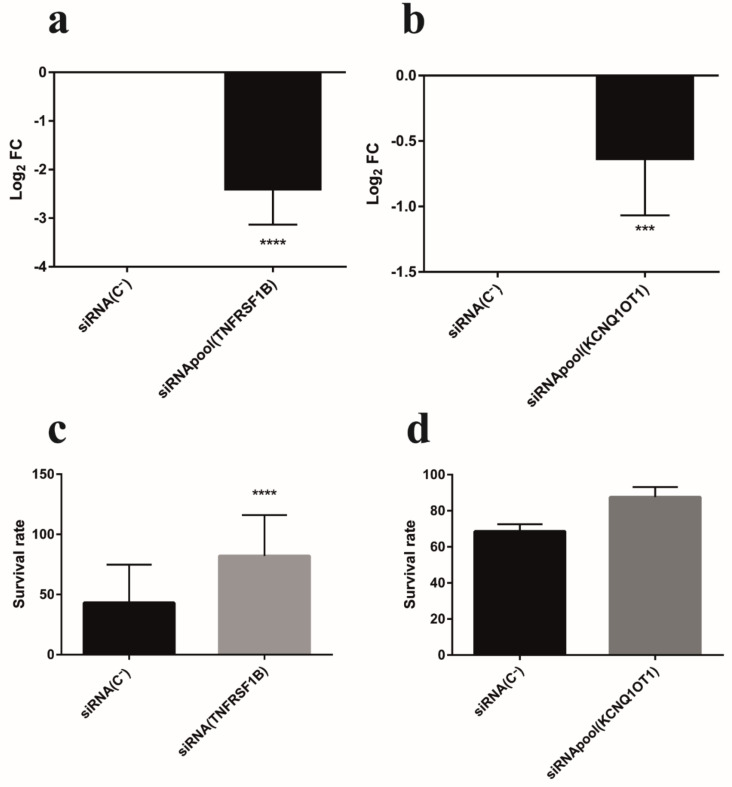
Functional validation of individual siRNA target transcripts: TNFRSF1B and KCNQ1-OT1. siRNA pools targeting TNFRSF1B (**a**), and KCNQ1-OT1 (**b**), reduce their respective transcript levels in 6CFSMEo- cells. Log_2_ FC values of 6CFSMEo- cells transfected with optimized siRNA pools, compared with control (6CFSMEo- cells transfected with a non-targeting siRNA sequence (siRNA(C-)). Data are expressed as means ± SD. *n* = 8, with three technical replicates/each. *** *p* < 0.001; **** *p* < 0.0001 compared to the control. Spectrophotometric quantification of the mean survival rate of the siRNA control non-target (siRNA(C-)) and the siRNA pools against TNFRSF1B (**c**), and KCNQ1-OT1 (**d**), transcripts in 6CFSMEo- cells in the presence of 1.2 mM H_2_O_2_ at 4 days after adding Alamar Blue reagent. Data are expressed as means ± SD. *n* = 5, with 8 Table 1. B; *n* = 2, with four technical replicates/each for KCNQ1-OT1. **** *p* < 0.0001, compared with the control.

Further supporting the participation of the above siRNA target gene catalog in the oxidative stress process entailed the demonstration of RNAi-mediated oxidative stress protection from airway epithelial cell damage through carefully designed siRNAs targeting sequences, unrelated to those unveiled from the randomized siRNA library, against defined siRNA target transcripts. Two selected transcripts were chosen for that purpose: KCNQ1OT1 and TNFRSF1B. The lncRNA KCNQ1OT1 is positioned in the KCNQ1 locus. Up to six different siRNA sequences, from the total of 167, matched within the *KCNQ1OT1* gene with a high percentage of homology (68–78%), indicating that this lncRNA might hold an important role in oxidative stress. Regarding TNFRSF1B, the corresponding siRNA clone presented a high percentage of matching homology (73%) with this transcript and induced significant resistance to oxidative stress (large effect size > 0.8). Moreover, TNFRSF1B may function as a death cell receptor [37], and the TNF-TNFR pathway is an important hub regulated by ROS in inflammation [38]. Thus, we set up to assess whether optimized pools of four different siRNAs could induce the silencing of KCNQ1OT1 and TNFRSF1B transcripts in 6CFSMEo- cells and, concomitantly, could generate the oxidative stress resistance phenotype previously obtained from the randomized siRNA library clones targeting KCNQ1OT1 and TNFRSF1B, respectively. In both cases 6CFSMEo- transfection of the respective siRNA pools, but not of a control non-targeting siRNA pool, was able to significantly downregulate these transcripts (Figure 4a,b). Moreover, these same siRNA pools induced oxidative stress resistance and increased the viability of 6CFSMEo- cells (Figure 4c,d). Analogous results were obtained using the non-CF airway epithelial cell line 16HBE14o- cells (data not shown).

**Figure 5:**
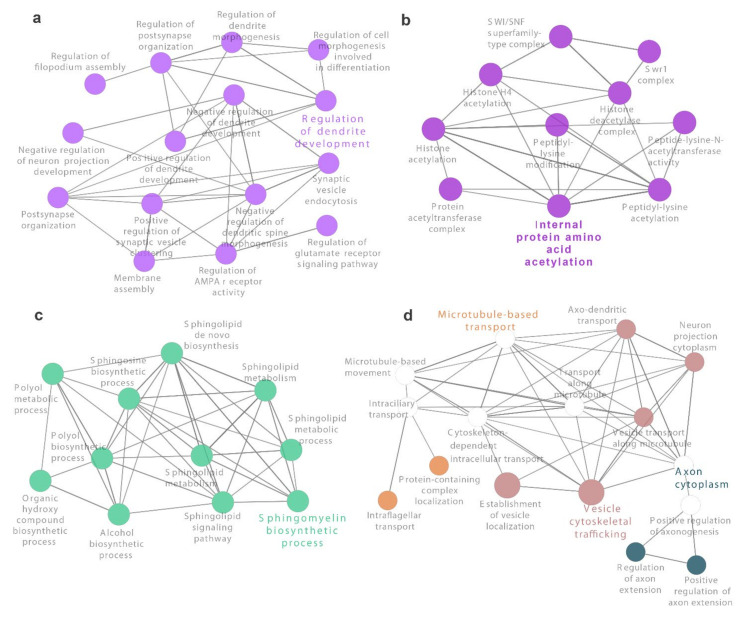
Main clusters of overrepresented biological pathways and terms from KEGG and Reactome knowledgebases. A total of 56 groups were identified with ClueGO Cytoscape plug-in from 152 enriched terms. Four out of the 56 groups are visualized. These correspond to clusters with 10 or more components. For each group, the most significant term is highlighted. (**a**) Regulation of dendrite development. (**b**) Internal protein amino acid acetylation. (**c**) Sphingomyelin biosynthetic process. (**d**) Microtubule-based transport, axon cytoplasm and vesicle cytoskeletal trafficking. Circle size is inversely proportional to obtained adjusted *p*-value.

No overrepresented pathways were found when analyzing the list of 444 siRNA target genes and independently considering KEGG, Reactome, or GO knowledgebases (Supplementary Info S2a). However, a total of 145 pathways and terms were enriched when integrating KEGG, Reactome, and GO knowledgebases. Clustering analysis uncovered 56 groups with four of them agglutinating 10 or more enriched terms. The most significant biological processes among the latter included “regulation of dendrite development” (GO:0050773), “sphingomyelin biosynthetic process” (GO:0006686), “microtubule-based transport” (GO:0099111) along with “vesicle cytoskeletal trafficking” (GO:0099518) and “axon cytoplasm” (GO:1904115), and “internal protein amino acid acetylation” (GO:0006475) (Figure 5 and Supplementary Info S2b).

To deepen the functional analysis of the 444 siRNA target genes, protein-protein interaction (PPI) networks were explored. Most of them (84%) were mapped to STRING database annotations (Supplementary Info S3). A first PPI network with very high confident interactions among siRNA target genes and direct interactors (1st shell) was composed by 125 genes, 26 (20.8%) of them belonged to the list of targets, and 357 interactions (Table 1).

**Figure 6:**
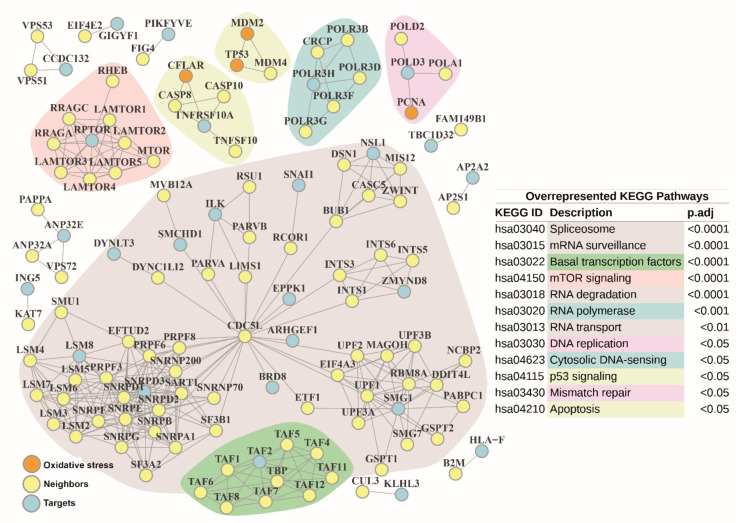
Protein-protein Interaction network built with siRNA target genes and 1st shell interactors from STRING database. Very high confidence 1st shell (direct) interactions were selected (minimum score of 900). Neighbors are highlighted in yellow. siRNA target genes are colored in blue. Genes related to oxidative stress are colored in orange. Main network submodules are marked with different background colors, according to the associated significantly enriched KEGG pathway obtained from the over-representation analysis (ORA) over the complete protein-protein interaction (PPI) network. Corresponding KEGG pathway identifiers (ID) and description are shown on the right. They are sorted by adjusted *p*-values retrieved from ORA.

Among the 125 genes, four (3.2%) were associated to oxidative stress: *CFLAR* (CASP8 and FADD-like apoptosis regulator) [39]; *PCNA* (proliferating cell nuclear antigen, activated by ROS [40], which plays a fundamental role in the regulation of oxidative stress) [41,42]; *MDM2*, and the tumor suppressor *TP53* (p53). The LCC was composed of 66 elements (52.8%) (Figure 6).

Interestingly, we found that the main hub gene of this LCC was the pre-mRNA splicing factor cell division cycle 5-like (CDC5L), involved in cell cycle regulation, immune surveillance, and DNA damage response [43,44], which was confirmed by a second PPI network that additionally included indirect interactors (2nd shell) (Figure S1). Alternatively, if interaction scores were relaxed to high confidence level, a third PPI network was expanded to 603 genes and 2,864 interactions when only considering direct interactors (Table 1). Among these, 115 (19.1%) genes belonged to the list of siRNA target genes, and 20 (3.3%) genes were annotated to the oxidative stress GO term (GO:0006979): *MDM2, PXN, TP53, CFLAR, HNRNPD, STX4, HNRNPM, STK25, CHUK, STK24, PCNA, PDK1, PDCD10, TNF, AKT1, ARNTL, FKBP1B, DNM2, HSF1*, and *MCL1*. Most of these were involved in focal adhesion, apoptosis, cellular senescence, regulation of actin cytoskeleton, and p53 signaling pathway. The corresponding LCC was composed by 395 (65.5%) genes (Figure S2).

Common overrepresented pathways and terms were identified after conducting ORA over the gene lists gathered from the above PPI (Figure 6) or the corresponding PPI LCCs (Figures S1 and S2) against knowledgebases (Table S3 and Supplementary Info S4a–d). Most of the common overrepresented KEGG pathways (80%) were annotated in the genetic information processing category, particularly related to: (i) transcription subcategory, with “RNA polymerase” (hsa03020), “spliceosome” (hsa03040), and “basal transcription factors” (hsa03022) pathways; (ii) translation subcategory, with “RNA transport” (hsa03013) and “mRNA surveillance” (hsa03015) pathways; (iii) folding, sorting and degradation subcategory, with “RNA degradation” (hsa03018) pathway; and finally, (iv) replication and repair subcategory, with “DNA replication” (hsa03030) and “mismatch repair” (hsa03430) pathways. Homologous processes in Reactome and GO were also retrieved. Interestingly, additional GO terms related to post-translation modifications such as ‘protein acetylation’ (GO:0006473) and epigenetic modifications such as ‘histone acetylation’ (GO:0016573) were also found. ORA also unveiled common significantly represented pathways related to intra- and extracellular cell communication and motility: “cell-extracellular matrix interactions” (R-HSA-446353), “cell-substrate junction” (GO:0030055), “regulation of expression of SLITs and ROBOs” (R-HSA-9010553), and “signaling by ROBO receptors” (R-HSA-376176), and their role in cell motility, “focal adhesion” (GO:0005925) and finally, “rho GTPases activate formins” (R-HSA-5663220). Other enriched, but non-common pathways, were “intraflagellar transport” (R-HSA-5620924) and “cillium assembly” (GO:0060271), both retrieved from 2nd shell PPI LCC, or “cell junction organization” (R-HSA-446-728) as part of cell-to-cell communication found in 1st shell PPI/LCC and more specifically, “gap junction trafficking” (R-HSA-190828) in 2nd shell LCC PPI. Further hits were associated to nervous system development processes, specifically, “CRMPs in Sema3A signaling” (R-HSA-399956) and “L1CAM interactions” (R-HSA-373760), both obtained from 1st shell LCC PPI. Overrepresented signaling pathways coordinating cell growth, proliferation, survival, apoptosis, and metabolism such as “mTOR signaling pathway” (hsa04150, R-HSA-165159 or GO:0031929), “PIP3 activates AKT signaling” (R-HSA-1257604), and “cell cycle arrest” (GO:0007050) were commonly found. Particularly for 1st shell LCC PPI, “energy dependent regulation of mTOR by LKB1-AMPK” (R-HSA-380972), “signaling by Hippo” (R-HSA-2028269), and “positive regulation of execution phase of apoptosis” (GO:1900119) processes emerged. In case of 2nd shell PPI, “MAPK family signaling” (R-HSA-5683057), “signaling by WNT” (R-HSA-195721), “endothelial cell apoptotic process” (GO:0072577) or “regulation of apoptotic signaling pathway” (GO:2001233) terms were also enriched. Finally, additional common enriched pathways involved “autophagy” (R-HSA-9612973) and “macroautophagy” (R-HSA-1632852) processes and “regulation of PTEN gene transcription” (R-HSA-8943724), the latter an event of previously mentioned “PIP3 activates AKT signaling”. All of them seemed to sustain a significant role in the outcome of oxidative stress in airway epithelial cell physiology.

### 3.4. Gene Targets from RNAi-Based Genome-Wide Screening Disclose Repurposable Drugs Able to Protect CF Airway Epithelial Cells from Oxidative Stress

The list of 444 siRNA targets conferring susceptibility to oxidative stress and the catalog of 488 additional direct interactors, at high confidence level, were employed to perform an exhaustive mining against public drug databases aimed to identify novel repurposable drugs to modulate CF airway epithelial cell damage against strong oxidative stress conditions.

From the DB database screening, 51 siRNA target genes were identified as drug targets, associated to 203 different drugs, 119 of which (58.6%) corresponded to approved drugs. Additionally, 82 first interactors were also identified as drug targets, being associated with 218 different drugs, and 82 of these (37.2%) corresponded to approved drugs (Supplementary Info S5, and Supplementary Info S6a–d). Interestingly, 14 drugs were in common between siRNA target genes and first interactors (Table 2).

From the TTD database screening, 22 siRNA target genes were identified as drug targets, associated to 161 different drugs, where five of them (3.1%) corresponded to approved drugs, and four of them (2.5%) to patented drugs. Moreover, 27 first interactors were identified as drug targets, being associated with 414 different drugs, and 31 of these (7.5%) corresponded to approved drugs, while 136 (32.9%) corresponded to patented drugs. No approved or patented drugs were in common between those retrieved from the siRNA target genes and those retrieved from the first interactors (Supplementary Info S5, and Supplementary Info S7a,b). Conversely, six drugs were found in common between DB and TTD mining: methoxamine and phendimetrazine (regarding siRNA targets), and imatinib, sirolimus, everolimus, and temsirolimus (regarding first interactors) (Table 2).

Examining the distribution of the genes/proteins targeted by the retrieved drugs from the PPI network analyses evidenced: (1) a handful of well-known genes/proteins that are targeted by multiple drugs, such as MTOR, TP53, or MDM2; and (2) uneven distribution of the targeting drugs among the different overrepresented pathways, with some of them being targeted by several drugs (e.g., in case of KEGG pathways, “DNA replication” (hsa03030), “p53 signaling pathway” (hsa04115), or “mTOR signaling” (hsa04150)), while others (e.g., “spliceosome” (hsa03040), “RNA polymerase” (hsa03020), “RNA transport” (hsa03013), and “RNA degradation” (hsa03018)) lacking approved drugs able to target any of their gene/protein components (Figure S3). Given this background, four candidate drugs from the above lists were chosen for further validation: methoxamine and everolimus (common approved drugs among both drug databases), and fostamatinib and zanubrutinib (common approved drugs between the siRNA targets and first interactors retrieved from the DB database). Since methoxamine is an α1-adrenergic receptor (ADRA1) agonist, we further selected a drug showing the opposite pharmacological activity, that is, the competitive ADRA1 antagonist doxazosin, inhibitor of smooth muscle contraction. Everolimus, akin to its analogs sirolimus and temsirolimus, is a well-known inhibitor of mTOR, a central regulator of growth and survival [45]. Finally fostamatinib, an cytosolic protein tyrosine kinase (Syk) inhibitor acting on several immunoreceptors [46], and zanubrutinib, a second-generation selective covalent BTK inhibitor acting on B cell receptors [47], are protein tyrosine kinase inhibitors also involved in cell growth and proliferation.

**Figure 7:**
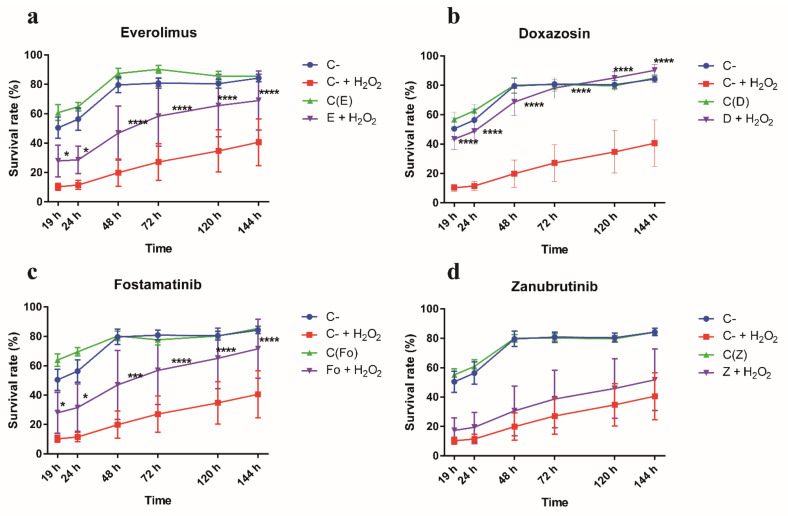
Selected drugs increased cell survival rate in CF airway epithelial cells. Alamar Blue cell viability assay at different times after strong oxidative stress induction. Spectrophotometric quantification of the mean survival rate of 6CFSMEo- cells under strong oxidative stress conditions in presence of the respective drugs. The drug working concentrations were: (**a**) everolimus (E; 5 μM), (**b**) doxazosin (D; 10 μM), (**c**) fostamatinib (Fo; 1 μM), and (**d**) zanubrutinib (Z; 10 μM); H_2_O_2_ was added at 0.9 mM. C-, untreated cells; C- + H_2_O_2_, untreated cells in presence of H_2_O_2_; C(X), cells treated with the respective drug; X + H_2_O_2_, cells treated with the respective drug in the presence of H_2_O_2_. Data are expressed as means ± SD; E: *n* = 5; D: *n* = 6; Fo: *n* = 6; Z: *n* = 2, with three technical replicates/each. * *p* < 0.05, *** *p* < 0.001, **** *p* < 0.0001 compared with control (C- + H_2_O_2_).

To assess whether these drugs would be able to confer oxidative stress resistance in CF airway epithelial cells, cell viability monitoring was performed over 6CFSMEo- cells either untreated or treated with the individual drugs and further exposed to a harmful H_2_O_2_ concentration (Figure 7). The working concentration for each individual drug was selected based in a preliminary viability assay to ensure lack of toxicity over the 6CFSMEo- cells. Overall, these results consistently suggested that everolimus, fostamatinib, and doxazosin at different degrees, but not zanubrutinib, were able to increase cell survival from strong oxidative stress conditions.

## 4. Discussion

The self-perpetuating infection-inflammation cycle occurring in the lungs of CF patients constitutes a chronic challenge to the integrity of airway epithelial cells, oxidative stress being a key element contributing to persistent cellular damage and preventing proper airway remodeling [2,48]. Indeed, oxidative stress is a complex process in which the excess of reactive oxygen species (ROS) affect, either directly or indirectly, all structural and functional components of cells at the molecular level. This ultimately impairs their function, promoting cell death and, thus, shaping essential aspects of cell biology and physiology impacting homeostasis and disease [38]. It has been postulated that antioxidant defenses play an essential role modulating the redox imbalance of the organisms. Nevertheless, little is known about the wide spectrum of molecules and pathways that influence oxidative stress outcome, particularly in CF airway epithelial cells facing a particularly hostile oxidative environment. In the present study, we performed a high-throughput, unbiased RNAi screening using a randomized siRNA library for the identification/discovery of known/novel genes and pathways involved in oxidative stress. Efficient knock-down of essential siRNA target genes should induce intrinsic cell protection from environmental oxidants in CF airway epithelial cells. Our randomized siRNA library encompasses multiple targets in every transcript from the human genome. Therefore, it is suitable for phenotypic cellular assays to identify genes whose expression is required for the screened phenotype without prior knowledge of their corresponding siRNA sequence(s). We employed the strong oxidant H2O2 for selecting CF airway epithelial cells whose knocked down transcript(s) conferred oxidative stress resistance and preserved cell viability. It is well known that ROS-mediated cell damage exhibits both apoptotic and necrotic cell death [49]. Moreover, human CF submucosal gland cells (6CFSMEo-) have been used as a surrogate to uncover the role of oxidative stress in CF epithelial integrity. In CF, lack of functional CFTR channels causes defective secretion by submucosal glands, the predominant site of CFTR expression in the human bronchus, leading to enhanced mucus production, persistent bacterial infection, and increased oxidative stress, which progressively damage the airways [50]. Moreover, submucosal gland progenitors can differentiate into the major airway epithelial cell types [51,52]. We performed a genome-wide loss-of-function pooled screen and subsequent stringent positive selection, monitoring 6CFSMEo- cell survival under a lethal dose of H_2_O_2_. This was followed by a consistent high-throughput individual validation step, uncovering a catalog of 167 siRNAs able to confer resistance to oxidative stress. Next, a rigorous homology-based computational methodology allowed inferring a reliable list of 451 6CFSMEo- transcripts as direct siRNA targets, which was complemented by retrieving its closest biomolecular interactions from the predicted interactome. Remarkably, we found that only four (near 1%) of the direct siRNA target transcripts/genes were associated to oxidative stress. All of them were involved in p53 signaling, cell cycle, and apoptosis pathways [53]. Therefore, this outcome stressed the relevance to decipher the contribution of novel oxidative stress response pathways critical for CF epithelial cell homeostasis, able to influence integrity/survival through adaptive changes in gene expression. Thus, different enrichment analyses unveiled a central role of splicing mediating the oxidative stress response in CF airway epithelial cells. Indeed, it has been shown that genotoxic stressors such as radiations and chemotherapeutic agents, and here oxidative stress, impact the alternative splicing or splicing efficiency of pre-messenger mRNAs in a general or gene-specific manner [54,55]. Stresses induce “non-productive” splice variants subjected to nonsense-mediated decay and, therefore, directly influence gene expression regulatory networks. Thus, splicing-mediated regulation of gene expression is faster than transcriptional regulation. Interestingly, genotoxic stressors seem to induce not only the pro-apoptotic splice isoforms of several genes such as members of the caspase and Bcl2 families [56,57], but also to regulate the splicing of MDM2 transcripts, which play a central role in the DNA damage response [58]. Consequently, a reduction of full-length MDM2 transcript levels protects p53 from degradation during stress exposure. Therefore, alternative splicing of both p53 [59] and p53 regulators may turn to be a widely employed mechanism in stress responses. In addition, alternative splicing seems to be influenced by other related overrepresented pathways in our study, related to the transcription machinery (e.g., “RNA polymerase” regarding transcription elongation [60]), and other epigenetic events: chromatin modifications such as “histone acetylation” [61,62] and “protein acetylation” of splicing factors [63]. In this context, it is intriguing that the PPI networks from our study have unveiled the splicing factor CDC5L as the most relevant hub-driving gene modulating splicing induced by oxidative stress in CF airway epithelial cells. In fact, CDC5L is a cell cycle regulatory element involved in the catalytic steps of mRNA splicing and DNA damage repair [44]. A recent study has shown that a bacterial stressor, the deadly pathogen *Klebsiella pneumoniae*, subverts host cellular machinery by targeting p53 and CDC5L as pivotal molecular targets with a profound role in disease pathogenesis [64]. Moreover, CDC5L has been postulated as a reliable biomarker in COPD, a major predisposing factor of which is oxidative stress [65]. Overall, the evidences from other studies [66] and ours seem to indicate that growth-inhibitory and death-promoting effects under strong oxidative stress conditions in CF airway epithelial cells could be mediated, at least partially, by the coordinate regulation of multiple genes at the level of alternative splicing. Therefore, therapeutic strategies targeted to the promotion or inhibition of splicing [67,68] may become useful to tackle the consequences of oxidative stress in CF airway epithelial cells.

Other relevant oxidative stress-sensitive aspects intrinsic of CF airway cell physiology that appeared significantly highlighted in our pathway analysis refer to cell remodeling, cell-cell communication, and motility. It has been recently shown that submucosal glands are required for normal antimicrobial activity and mucociliary transport, two key host defenses protecting the lung that are disabled in CF patients [69]. Thus, it is not casual that related pathways such as intraflagellar transport and cilium assembly are significantly overrepresented in our study, because they may impact airway epithelial cell integrity upon repeated oxidative stress challenges experienced by CF patients. Submucosal glands are innervated by vagal cholinergic efferents [70,71], enabling them to rapidly secrete submucosal gland products, such as antimicrobials and mucins, on demand for a proper response to acute pathogen challenge. Moreover, strands of mucus produced by submucosal glands bind to large particles and transmit forces to beating cilia [72], whose function is impaired in CF. Our results suggested that, while dysfunctional CFTR generates an electrolyte imbalance directly influencing mucus viscosity in the airway epithelia [73], oxidative stress may impact cilia structure and function, particularly the intraflagellar transport (IFT) machinery, leading to impaired intraciliary transport of large protein complexes along microtubules and cilium assembly [74,75]. In fact, ROS, particularly H2O2, produced by polymorphonuclear leukocytes in response to infection decrease the beating frequency of respiratory cilia [76]. Moreover, defective ciliogenesis results in chronic upper airway infection and progressive deterioration of lung function [77,78]. Alternatively, considering the high plasticity of airway epithelial cells following injury [11], oxidative stress may affect not only repair mechanisms, but also airway epithelial cell development programs regulating, among others, cell movement, ciliogenesis, and cilia function. Relatedly, our protein network analysis highlighted the susceptibility of cell guidance pathways playing an essential role in cell remodeling, such as “regulation of expression of SLITs and ROBOs”, “signaling by ROBO receptors”, and “CRMPs in Sema3A signaling”, to oxidative stress. Slit2 has been deemed essential for airway epithelial cell apical surface insertion and expansion [79]. Moreover, Sema3A has been shown to negatively regulate lung morphogenesis [80]. To note, the “signaling by Hippo”, which promotes regeneration and remodeling of the airway epithelium [81], seems also to be disturbed under strong oxidative stress conditions.

Furthermore, as observed through our pathway analysis, CF airway epithelial cells quickly responded to oxidative stress through general responses (inhibition of cell proliferation and/or induction of cell death through apoptosis related pathways) and metabolic adaptations such as DNA damage repair. The tumor suppressor PTEN has been shown to hold an essential role in both processes, inducing apoptosis and regulating the PI3K/AKT pathway, and controlling the DNA damage response through its interaction with the Chk1 and p53 pathways [82]. In fact, the PI3K/AKT/mTOR pathway regulates oxidative stress-related senescence. Inhibition of this pathway either endogenously (e.g., through AMPK) or exogenously (e.g., by rapamycin), extends the lifespan from yeast to mammals through the control of cell metabolism and autophagy [83,84,85].

Finally, public drug database mining considering the oxidative stress-susceptible gene set obtained from the RNAi screen uncovered candidate approved and, therefore, repurposable drugs able to protect CF airway epithelial cells from the harmful outcomes of oxidative stress exposure. Indeed, anti-oxidant therapy, even in CF patients that have an impaired absorption of dietary antioxidants in the gut and are unable to efflux glutathione, has not met expectations up to now [10,31,86]. Moreover, a recent study showed that CFTR correctors and antioxidants partially normalize lipid imbalance but not the abnormal basal inflammatory cytokine profile present in CF bronchial epithelial cells [87]. Bearing in mind the above-outlined underlying molecular mechanisms involved in oxidative stress-mediated damage over CF airway epithelial cells, we validated three clinically approved drugs that confer significant oxidative stress resistance in CF airway epithelial cells: the mTOR inhibitor everolimus [88], the α-1 adrenergic receptor antagonist doxazosin [89], and the spleen tyrosine kinase (Syk) inhibitor fostamatinib [90]. Similar to other mTOR inhibitors, everolimus targets mTORC1 and not mTORC2. mTOR activity is upregulated in CF bronchial epithelial cells. Moreover, inhibition of the PI3K/AKT/mTOR pathway induced increased CFTR stability and expression by restoring autophagy [91]. Activation of the mTORC1/PGC-1 axis promotes mitochondrial biogenesis and induces cellular senescence in the lung epithelium [92]. Conversely, a potent PI3K/mTOR inhibitor showed anti-fibrotic activity in idiopathic pulmonary fibrosis [93]. Doxazosin causes smooth muscle relaxation and is used to treat hypertension and benign prostatic hypertrophy. Na+-Cl- co-transport in human airway epithelium is regulated by α-adrenergic receptors [94]. Moreover, a recent study indicated that modulation of α-adrenoceptor signaling protects against photoreceptor cell death in retinal detachment by inhibiting oxidative stress and inflammation [95]. On the other hand, doxazosin has been postulated as an antagonist of the cell guidance signaling complex ephrin-ephrin receptor [96]. Fostamatinib is an ATP-competitive inhibitor of Syk, a member of the Src family of non-receptor tyrosine kinases, which associates directly with surface receptors and is involved in a variety of signal transduction pathways and diverse biological functions [97]. Syk has been proposed as a novel target for treatment of inflammation in lung disease [98] and, recently, as a target therapy for *P. aeruginosa* infection [99,100]. Furthermore, topical Syk inhibition in the lung, without systemic exposure, has proven sufficient to inhibit an early asthmatic response in rats [101]. Nevertheless, the clinical effects of fostamatinib are likely mediated by inhibition of several Syk-dependent and Syk-independent signaling pathways. Interestingly, a recent high-content screen for mucin-1-reducing compounds has identified fostamatinib as a repurposing drug candidate for acute lung injury [102]. Thus, compared with traditional drug development schemes, the above approach provides a quick and cost-effective procedure for the rational identification of novel airway cell “protective” medicines to surmount oxidative stress and the concomitant inflammatory response striking CF patients, particularly those insensitive to potentiators and correctors. Nonetheless, further mechanistic studies are necessary to define the overall contribution of the above-disclosed oxidative stress susceptibility genes and pathways in more complex pathophysiological settings such as CF organoids, or CF animal models for in vivo validation, followed by carefully designed clinical trials to assess the therapeutic efficacy of the repurposed drug candidates.

## 5. Conclusions

In our study, we showed the usefulness of combining unbiased genome-wide knock-down to uncover new genes/pathways involved in oxidative stress with data mining through public drug databases to identify and characterize novel repurposable drugs. These, either alone or combined with the novel CFTR modulators, may benefit CF patients preserving airway epithelial cell integrity and preventing accelerated lung function decline. This strategy could also improve the outcome of other respiratory pathologies in which oxidative stress takes a central stage.

## Figures and Tables

**Table 1:** Main features of obtained protein-protein interaction (PPI) networks.

Feature *		Adding Direct Interactors (1st Shell)		Adding Indirect Interactors (2nd Shell)
	Only siRNA Targets	High Confidence Interactions	Very High Confidence Interactions	Very High Confidence Interactions
Total genes	14	603	125	650
siRNA target genes	14 (100%)	115 (19.1%)	26 (20.8%)	26 (4.0%)
Total interactions	8	2864	357	1497
Oxidative stress genes	0 (0%)	20 (3.3%)	4 (3.2%)	20 (3.1%)
LCC size	3 (21.4%)	395 (65.5%)	66 (52.8%)	628 (96.6%)

* Four different PPI networks are listed. Main features refer to total genes included in the network with at least one interaction, which of them corresponded to siRNA target genes or oxidative stress-related genes, the total number of network interactions and the largest connected component (LCC) size. Absolute and relative (%) number of genes are specified. Relative values refer to the number of total genes in the corresponding network.

**Table 2:** Common approved drugs between the siRNAs targets and interactors from the “Drug Bank” (DB) database, and among both “Drug Bank” (DB) and “Therapeutic Target Database” (TTD).

Drug Name	DB Drug Code	TTD Drug Code
Pyridoxal phosphate	*DB00114*	-
Amitriptyline	*DB00321*	-
Epinephrine	*DB00668*	-
Amrinone	*DB01427*	-
Dalfampridine	*DB06637*	-
Zanubrutinib	*DB15035*	-
Tiludronic acid	*DB01133*	-
Zinc	*DB01593*	-
Zinc acetate	*DB14487*	-
Zinc chloride	*DB14533*	-
Zinc sulfate	*DB14548*	-
Fostamatinib	*DB12010*	-
Citric acid	*DB04272*	-
Copper	*DB09130*	-
Methoxamine	*DB00723*	D09GYT
Phendimetrazine	*DB01579*	D0T6SU
Imatinib	*DB00619*	D0AZ3C
Sirolimus	*DB00877*	D03LJR
Everolimus	*DB01590*	D0K3QS
Temsirolimus	*DB06287*	D0ES1Q

## Data Availability

Data is contained within the article and Supplementary Materials.

## Supplementary Materials

The following are available online at https://www.mdpi.com/article/10.3390/antiox10121936/s1, Figure S1: Protein-protein Interaction network built with siRNA target genes, 1st and 2nd shell interactors from STRING database; Figure S2: Protein-protein Interaction network built with siRNA target genes and 1st shell interactors from STRING database; Figure S3: Protein-protein Interaction network built with siRNA target genes and 1st shell interactors from STRING database including associated drugs; Table S1.- Primer sequences for randomized siRNA library generation/screening, and pre-designed siRNAs directed against the TNFRSF1B and KCNQ1OT1 transcripts; Table S2 List of the final 167 siRNA sequences obtained through randomized siRNA library screening in 6CFSMEo- cells; Table S3. Number of overrepresented pathways and terms from the built protein-protein interaction (PPI) networks or corresponding Largest Connected Components (LCC). Common overrepresented pathways among the three analyzed cases are quantified. Supplementary Info S1: siRNAs Targets Functional Annotation; Supplementary Info S2a: ORA only siRNA targets; Supplementary Info S2b: Clue GO Result Table; Supplementary Info S3: PPIs Interactions List; Supplementary Info S4a: ORA Common Overrepresented Pathways PPIs; Supplementary Info S4b: ORA PPI 1stShell complete th 900; Supplementary Info S4c: ORA PPI LCC 1stShell th 700; Supplementary Info S4d: ORA PPI LCC 2nd Shell th 900; Supplementary Info S5: DB TTD Raw siRNAs targets and Direct Interactors; Supplementary Info S6a: List Of Approved Drugs siRNAs Targets DB; Supplementary Info S6b: List Of Approved Drugs Interactor Genes DB; Supplementary Info S6c: List Of Drug Targets Appr Drugs siRNA Targets DB; Supplementary Info S6d: List Of Drug Targets Appr Drugs Interactors DB; Supplementary Info S7a: Appr & Pat Drugs w Targets siRNA Targets TTD; Supplementary Info S7b: Appr & PatDrugs w Targets Interactors TTD.
