# Supplementary material for: Genome-Wide RNAi Screening Identifies Novel Pathways/Genes Involved in Oxidative Stress and Repurposable Drugs to Preserve Cystic Fibrosis Airway Epithelial Cell Integrity
Source: Antioxidants (Basel). 2021 Dec 2;10(12):1936. doi: 10.3390/antiox10121936 (PMC8750174; doi:10.3390/antiox10121936)
Supplement: Supplementary file 1 [file antioxidants-10-01936-s001.zip › SUPPLEMENTARY TABLES_(ARAN) (08-11-2021).pdf]

Supplementary Tables

Table S1. Primer sequences for randomized siRNA library generation/screening, and pre-designed siRNAs directed against the TNFRSF1B and KCNQ1OT1 transcripts.

**Primer Sequences**		
**Name**	**Sequence (5' → 3')**	**Number of Nt**
ClaI/NotI F	CCGCGCATCGATGCGGCCGCCAAGGTCGGGCAGGAAGAGGGCCTA	45
EcoRI-H1 R	CGCGAATTCGAACGCTGACGTCATCAACCCGCTCCAAGGAATCGC	45
BamHI-MluI-siRNA(EGFP)-SmaI R	GCGCGGATCACGCGTAAAAACTTCAGGGTCAGCTT-GCCGTTTTTCCCGGGCTTTCCACAA	60
pLIB-N R	TGTGGAAAGCCCGGGAAAAAGNNNNNNNNNNNNNNNNNNTTTTTAC-GCGTGTCTCATACAGAACTTATAAGAT	73
pCI-neo(CMV$^-$)LIB F	TTCAAGAACTCTGTAGCACCGCCT	24
pJET 1.2 F	CGACTCACTATAGGGAGAGCGGC	23
pJET 1.2 R	AAGAACATCGATTTTCCATGGCAG	24

**Pre-designed siRNAs**	
**siRNA name**	**Sequence (5' → 3')**
ON-TARGET plus Non-targeting siRNA (C-)	UGGUUUACAUGUCGACUAA
**TNFRSF1B**	
ON-TARGET plus SMART pool siRNA J-003934-05 (siRNA1)	CGACUUCGCUCUUCCAGUU
ON-TARGET plus SMART pool siRNA J-003934-06 (siRNA2)	GGAAUGUGCCUUUCGGUCA
ON-TARGET plus SMART pool siRNA J-003934-07 (siRNA3)	CAUCAGACGUGGUGUGCAA
ON-TARGET plus SMART pool siRNA J-003934-08 (siRNA4)	AGCCUUGGGUCUACUAAUA
**KCNQ1OT1**	
Lincode SMART pool siRNA N-188025-01 (siRNA1)	GCCAAGAACCAACGAAUGA
Lincode SMART pool siRNA N-188025-02 (siRNA2)	GCAUAUCUGUCUUCCGUAU
Lincode SMART pool siRNA N-188025-03 (siRNA3)	GAAUACAGAGAACGGCUGA
Lincode SMART pool siRNA N-188025-04 (siRNA4)	GAAUACAUAAAGCACGAUU

Table S2. List of the final 167 siRNA sequences obtained through randomized siRNA library screening in 6CFSMEo- cells.

**siRNA_id ***	**siRNA_seq (5' → 3')**	**siRNA_id**	**siRNA_seq (5' → 3')**
1	GTATACCCTGTACTAGTTG	92	GAGGGTGATGTTTGCGTGG
2	GGCATTGGCCTATTGGTAT	93	GGGATCGATACCATTACTT
3	GAGCGAGCTACGGGACTCT	94	GGCTCTGTGTATGTAAGTC
4	GTGGGCCCAGCGTACTTTC	95	GGCTGGTCATAACCTCTCT
5	GCGTTATGGTGTTGGTAAC	96	GACGTGAACGAAAGACTTA
6	GGTTAATCAAATTGGTGGA	97	GGATCGATCTACGCACAGT
7	GCGCACGGGGCGCTAGTTT	98	GGTTCTTCGTACTATTGGC
8	GGGTGCGTGGGCAGTGTGT	99	GTTAAGCATAGGATGATAA
9	GTGAGAGTGAGCGTTTATC	100	GCACATGAGGGATGCAACA
10	GTGACTTGCTTGGTACTTC	101	GGTTGCTGCGAGCTGCCAC
11	GAGCAACATACCAGTGTCG	102	GAGAGTCGGAGGTACTTGC
12	GGCTCGTCACCCGTGAATC	103	GACTTTAGGACGTGCATCG
13	GAGTCTAGATAGTGTTTT	104	GCACTTTATGTTCCGCTCC
14	GTGCCAGTAAGGCCGTACC	105	GGCCGGTCGGTTCTCGGTC
15	GTCGTGCTAATGAGGATTA	106	GTATTAAGGGCGATCCGTA
17	GGCGATAGAGTTGTGCGCG	107	GGGTTGCACCGCTTTCTAC
18	GTTTGCTGGCTACTCCTTT	108	GCGTGGTTCACGCTGTCCA
19	GAGGCTTGCGTGCTGGACC	109	GATGCTTGCAAGAATAATT
20	GTGTTTTCCATGTGCTTTA	110	GGGTCATGGGGGCTGCGCA
21	GCATCTGTCATGCATACTC	111	GGTCTGTGCAACCAACAAT
22	GTCGGTATGGCTAGTTACG	112	GGTCTATTTGGCATAGTAT
23	GTATGGTCAACACAGTTCC	113	GTGAGATGTAGCATATCTG
24	GCGGCAAGCTGACCCTGAA	114	GACCCGTACGTGTCAGATC
25	GTTTAAGGTTGCAGTT	115	GTGAGGGTTAGTCTCGTTC
26	GGCAATGATACTTATCCTA	116	GGCATATGGTCGGTCTGGT
27	GGGTTACTTATCATGAACC	117	GCACGTCTCGGAGCAGGTG
28	GTCGCAGTCTGCATTTTGC	118	GATCGCCACACGATTCGCT

29	GTCTTCGCCATGATTCTGG	119	GGATGGTCCTTAATCGTTA
30	GGCTCGACTGAGGTGTGGA	120	GTCACGCCGCTACGCACCG
31	GCGATACCTCCCTACGCC	121	GGATGTCGTATATCTGGGA
32	GTCTGAGAAATACTTTCGA	122	GTTGCTGGGAGCCTTTCCC
33	GATAGTCTGGGCCAATATC	123	GGGTAATTGAGTCGACGTC
34	GCGGTTTGCGATTGGGCGG	124	GAACCGGTCTGGCTACAAC
35	GGTGCCACGATATCTGAAG	125	GTAACGTGGCGCGGGTACA
36	GGTGTGTGTTTTCTACCTT	126	GATGCAACCTGGTTTTTAC
37	GGCGTGTCGGGATGTTCCC	127	GGCGCTAAGGTAGCCTCAT
38	GGCAACGCTGTGGTAATAC	128	GACGCTGCCCGCCCACCGT
39	GCATTTTCGTCTTCTTGGG	130	GACAGGATAGGATTCCTTC
40	GCTCGCGCGAAATGAGTGA	131	GGAACAATGGTCTCATGTC
43	GCGCGACGGGTCTTATCGT	132	GGCCAGCCCTCGGCTTCGT
44	GATATCTTGTGCCTGTCGT	133	GACGGACCGAATTTACGTG
45	GCGTCTTTTTGGAATGCGC	134	GATGCTACGGGTTTGGTAT
47	GGTGATCTGCTGGCTTACT	135	GAATGACAGGGTCAGTATA
48	GGCTCAGTGGATTATCATT	136	GGAAGGCCAGTTTGCGTTG
49	GTTGCTCCAACATTACATG	137	GATAGAAGTGGTATTTGGC
50	GTTGACAGGAACATAATAA	138	GTGTGGGATTATCAAAACC
51	GTGCTGGTTCAAATAGTTT	139	GACGTCATGGGTTTCCGTT
52	GGTCTTATCAACGTCATGC	141	GCCCTCTCCCTTTGAGCCA
53	GCCTAATAGTGTAGTCAAC	142	GCGAATGGGAATTTCCTCT
54	GTGCCAGAATGTGGTGCAC	144	GGCAACAAAGAGGTAATAC
55	GGAATGAGGGGACGGAGAG	145	GCGGGTTTGTGTCTACTGT
56	GGGTGTTCAAACAAATAAG	146	GTCTGCCTCACTTACTATA
58	GATAAGCACCCCAATTGAG	147	GGGGAGGGCATGTTATAGA
59	GTGTTTCCGGGGGTAAGGA	148	GGTATGACGGCGTGATCAA
60	GCTGGTTCCGTCTTCTCAT	149	GCCAAGGCACCCC
61	GTGGAAATTTCGAGTCGCG	150	GGCCAATAGGGTATCGCAC
62	GCTCGCCTGGTCGGACAGC	151	GGTGACAATACAGTTCATA
63	GTTGGGTGTACTGGTCCCT	152	GAGTGACCGCGCACAATGC
64	GCGTGCGATGTTTACAGTG	153	GATCGCTTGATGGGCTAGG
65	GGAAGTGGACTCACCCCTT	154	GCGGCCAGCTCCTCTATGC
66	GGGCATGAACAGTCCTTAG	155	GGTTGGTGGGTGGGACGCG
68	GCGCGTGTCCTTACGTTTT	156	GATACATTGGTTTTCGCCC
69	GCGTAATTTGGGGATACGA	157	GCGCTACTTACGGTCTTAT
70	GACTGCTTCTATTTGACAA	159	GACGCCGTAAAGTGCCCGA
71	GTAGCAACTCTGGCGTAGT	160	GTGTAAGCTATTTGTGTAC
72	GGTCGGCAGTAAGCAGTGA	161	GTGGTATCTCTGGTTCCTC
73	GCCCATTCGCTCTTGTTCA	162	GGTTACCGCGTCATCTATG
74	GGCTCCTTACGCGTAATGC	163	GTAGTAGAGATGTAAGTGG
75	GGTAAATCTTCCCTATCGG	164	GTGTCATTCATCTCGTGTG
78	GTGCGTGGGTAGCTCTTTC	165	GCTTACGTTTTGTGCTATG
79	GTGACACAGCCTACTAGCT	167	GGACGCTTCGTGCGAGACG
80	GGTGGTAATGAGGTCGTCT	168	GTATTACCGTGTCTTTATC
81	GGTCCGCAACACCGCGATA	169	GAGCTTTAGTCGCGTGTCT
82	GCCTCATGGCCAACATGTC	170	GGTGGTCCCTCCCTCCTCC
83	GCGTGATGGGCCCTAAATG	172	GTTGTCCTTCTGCCCAGAA
84	GGGAGGGCTGGTCCTATGT	173	GTTGTCCTCACCGGTATTA
85	GGTTGTAGCTCAATTGTGT	174	GTTAAACCACTGAGATGGT
86	GGCTGACCAGTCCGCTTTT	175	GGTAGGACCGAAAACGTAT
87	GCTGGATGTCAGTCTATAT	176	GGAGTGTATGTTGCTATCG
88	GGTCTCAATACGCTTCTGT	177	GGATTTAGGCTCCCGTCC
89	GGGGATACAAAGTCCTCGT	178	GCAAGGTCGGTAAGTGTCT
90	GGAGGACGTGCTTCGGGGA	179	GGTGTTGTGCTTGCCTCTG
91	GTTTTGCCTAACGTGTCGT	180	GGTTCACGCGCGAAATCGT
		181	GGAGTAAAGATACTATCGC

* Clones siRNA 46 and 171 were discarded because they did confer no or marginal survival, respectively, compared to control pEGFP-N1-transfected cells. Clones 129, 143, and 158 and 166 were further discarded because their complete target alignment was < 68 % and/or they presented a centered mismatch with its homologous target. Clones 16, 41, 42, 57, 67, 76, 77, and 140 had no effect size.

Table S3. Number of overrepresented pathways and terms from the built protein-protein interaction (PPI) networks or corresponding Largest Connected Components (LCC). Common overrepresented pathways among the three analyzed cases are quantified.

Database.		Adding Direct Interactors (1st shell)		Adding Indirect Interactors (2nd shell) Very High Confident Interactions*	Common Overrepresented Pathways or Terms
		High Confident Interactions*	Very High Confident Interactions		
**KEGG Pathways**		**52**	**15**	32	10
**Reactome**		258	123	442	91
**The Gene Ontology**	Biology Process	423	203	474	137
	Molecular Function	89	64	132	30
	Cellular Component	167	102	215	75

* Overrepresented pathways and terms were obtained from the list of genes composing the LCC for the different explored PPIs (FDR adjusted *p*-value < 0.05). KEGG Pathways, Reactome and The Gene Ontology databases were independently considered.
