# Supplementary figures and images for: Genome-Wide RNAi Screening Identifies Novel Pathways/Genes Involved in Oxidative Stress and Repurposable Drugs to Preserve Cystic Fibrosis Airway Epithelial Cell Integrity
Source: Antioxidants (Basel). 2021 Dec 2;10(12):1936. doi: 10.3390/antiox10121936 (PMC8750174; doi:10.3390/antiox10121936)

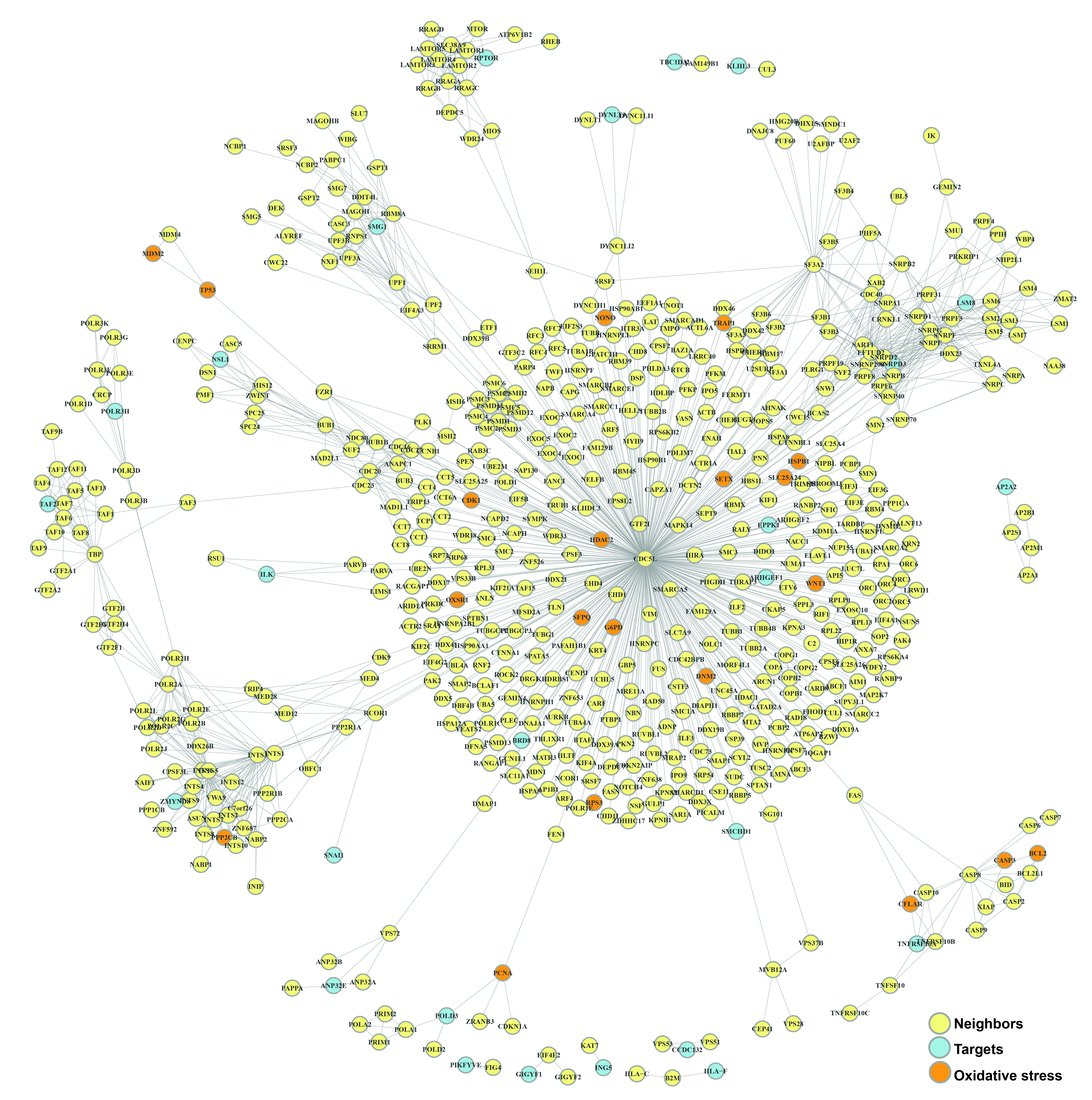

Supplement: Supplementary file 1 [file antioxidants-10-01936-s001.zip › FIGURE S1.tif]

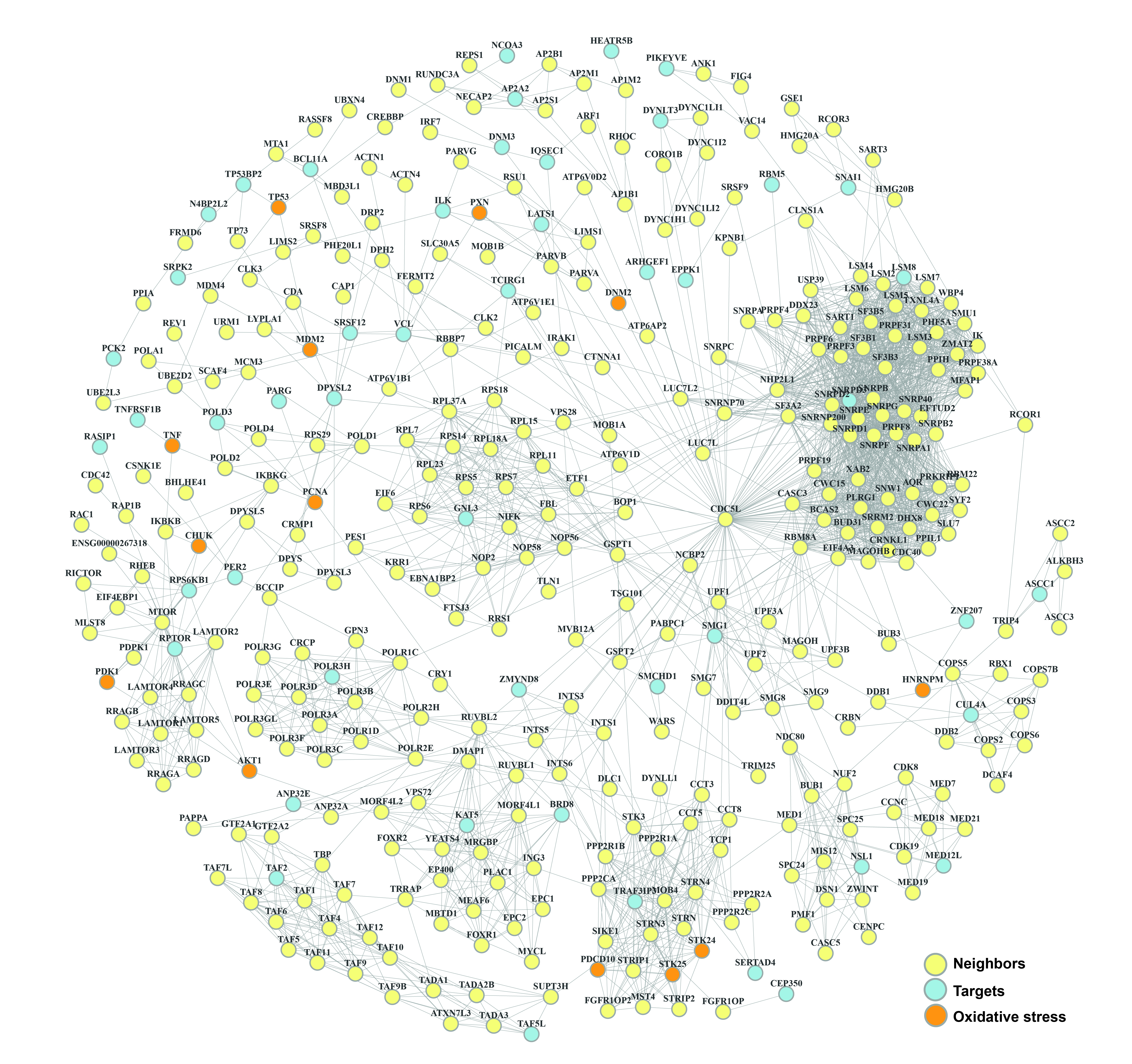

Supplement: Supplementary file 1 [file antioxidants-10-01936-s001.zip › FIGURE S2.tif]
